# PPAR*α*/*γ*-Independent Effects of PPAR*α*/*γ* Ligands on Cysteinyl Leukotriene Production in Mast Cells

**DOI:** 10.1155/2008/293538

**Published:** 2008-11-09

**Authors:** Masamichi Yamashita

**Affiliations:** Laboratory of Food Science, Department of Bioresource Science, Nihon University Junior College, 1866 Kameino, Fujisawa 252-8510, Japan

## Abstract

Peroxisome proliferator-activated receptor (PPAR) *α* ligands (Wy-14,643, and fenofibrate) and PPAR*γ* ligands (troglitazone and ciglitazone) inhibit antigen-induced cysteinyl leukotriene production in immunoglobulin E-treated mast cells. The inhibitory effect of these ligands on cysteinyl leukotriene production is quite strong and is almost equivalent to that of the anti-asthma compound zileuton. To develop new aspects for anti-asthma drugs the pharmacological target of these compounds should be clarified. Experiments with bone-marrow-derived mast cells from PPAR*α* knockout mice and pharmacological inhibitors of PPAR*γ* suggest that the inhibitory effects of these ligands are independent of PPARs *α* and *γ*. The mechanisms of the PPAR-independent inhibition by these agents on cysteinyl leukotriene production are discussed in this review.

## 1. INTRODUCTION

Asthma is defined as “a common chronic disorder of the airways that is complex and characterized by variable and recurring symptoms, airflow obstruction, bronchial hyperresponsiveness, and an underlying inflammation” [1]. Many types of inflammatory cells, neutrophils, eosinophils, lymphocytes, and mast cells contribute to the development of asthma.

Mast cells are differentiated from bone marrow stem cells and release various mediators of inflammation, such as histamine, through degranulation and arachidonic acid metabolites through *de novo* synthesis in response to pathological stimuli in asthma, atopic dermatitis, and other conditions. Immunoglobulin (Ig) E, a protein from B lymphocytes, increases in the serum of patients with type I allergic diseases [2].

**Figure 1:**
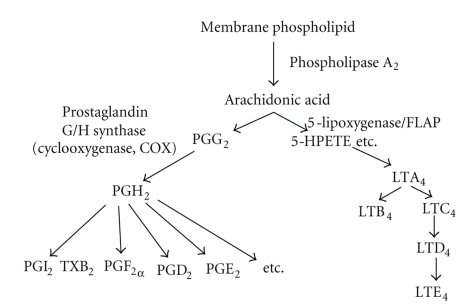
Diagram of arachidonic acid metabolism.

**Figure 2:**
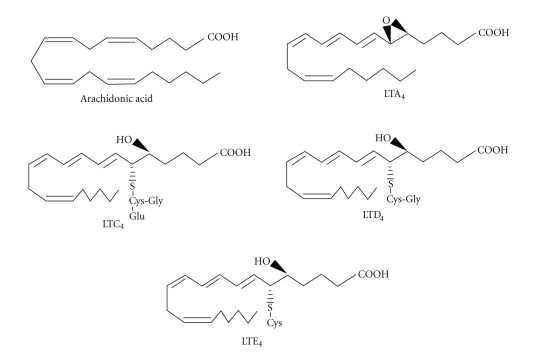
Chemical structures of arachidonic acid and cysteinyl leukotrienes.

Arachidonic acid is metabolized into many biologically active lipids, such as prostaglandins via cyclooxygenase, and leukotrienes (LTs) via 5-lipoxygenase (5-LOX). Arachidonic acid liberated from membrane phospholipids by phospholipase A_2_ is then metabolized into LTA_4_ by the 5-LOX/5-LOX activating protein (FLAP) complex (Figure 1). LTA_4_ is metabolized into LTC_4_ by conjugating cysteine, glycine, and glutamic acid via LTC synthase [3]. LTC_4_ is subsequently metabolized into LTD_4_ and LTE_4_ via the contribution of dipeptidases [4] or cytochrome P450 [5] by glutamic acid and glycine degradation (Figure 2). The LTs C_4_, D_4_, and E_4_ are called cysteinyl LTs (cysLTs) because they contain cysteine in their molecules. The cysLTs are regarded as main mediators of asthma because of their potent constricting effects on bronchiolar smooth muscle [6]. Specific receptors of cysLT are known [7, 8], and the inhibitors of the receptor [9] and the inhibitors of 5-LOX/FLAP activity [10–12] have been used to treat asthma.

Peroxisome proliferator-activated receptors (PPARs) are a family of transcription factors that are part of the nuclear receptor superfamily. The PPARs have 3 subtypes from the independent genes *α*, *β* (also called *δ*), and *γ*. A group of hypolipidemic agents, such as clofibrate and fenofibrate, are known to be ligands for PPAR*α*, and some agents used to treat type 2 diabetes mellitus, such as rosiglitazone, pioglitazone, and ciglitazone, are known to be ligands for PPAR*γ*. Some physiological fatty acids, such as leukotriene B_4_ and 15-deoxy-Δ^12‐14^ prostaglandin J_2_, are reported to be ligands for PPAR*α* and PPAR*γ*, respectively [15, 16].

## 2. LIGANDS FOR PPAR*γ* INHIBIT cysLT PRODUCTION IN MAST CELLS

Troglitazone (1 *μ*M), a PPAR*γ* ligand formerly used to treat type 2 diabetes mellitus, inhibits LTB_4_, LTC_4_, and LTE_4_ production induced by the type I allergy mechanism in a mast cell line, RBL-2H3 [17]. The inhibitory effects of troglitazone on these LTs are strong and similar to those of the clinically-used 5-LOX inhibitor zileuton (1 *μ*M) [17]. Another PPAR*γ* ligand, ciglitazone (30 *μ*M), also inhibits LTC_4_ production [18]. Neither troglitazone nor ciglitazone affects hexosaminidase release, the index for mast cell degranulation, or prostaglandin D_2_ production via cyclooxygenase [17, 18]. The observations that 0.1 *μ*M of the PPAR*γ* antagonist GW9662, which inhibits the PPAR*γ* activation of (AOx)_3_-TK-Luc promoter induced by the PPAR*γ* ligand rosiglitazone [19], did not affect LTC_4_ production [18] and that 30 *μ*M of GW9662 inhibits LTC_4_ production (our unpublished data) in the IgE-sensitized, and Ag-treated RBL-2H3 mast cell line obscures the contribution of PPAR*γ* on LT production in mast cells.

## 3. LIGANDS FOR PPAR*α* ALSO INHIBIT cysLT PRODUCTION IN MAST CELLS

Whether PPAR*α* ligands affect LT production in mast cells has been examined, and the PPAR*α* ligands fenofibrate (100 *μ*M) and Wy-14,643(30 *μ*M) have been reported to inhibit calcium ionophore A23187-induced cysLT production by the RBL-2H3 mast cell line [13]. However, Wy-14,643 does not significantly inhibit cysLT production by the IgE-sensitized and Ag-treated RBL-2H3 mast cell line. Neither fenofibrate (100 *μ*M) nor Wy-14,643 (30 *μ*M) affects radioactivity released from the IgE sensitized [^3^H]-arachidonic acid prelabeled RBL-2H3 mast cell line following treatment with Ag, which is an index of arachidonic acid release from mast cells. Neither fenofibrate (100 *μ*M) nor WY-14,643 (30 *μ*M) affects lipid peroxidation, which is an index of 5-LOX activation, whereas troglitazone (1 *μ*M) and zileuton (1 *μ*M) strongly inhibit lipid peroxidation [13].

## 4. ARE THE INHIBITORY EFFECTS OF THESE PPARs LIGANDS VIA PPARs?

**Figure 3:**
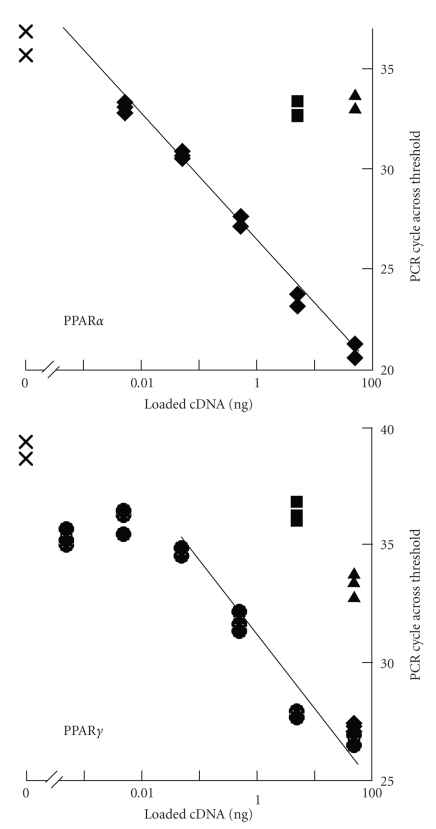
Measurement of mRNA levels of PPAR*α* (upper panel) and PPAR*γ* (lower panel) with real-time semiquantitative PCR. Total RNA (1 *μ*g) extracted from white adipose tissue (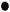), liver (▲), BMMC (■), and RBL-2H3 mast cells (♦) was supplemented with 50 pg of chloramphenicol acetyltransferase RNA and then reverse-transcribed. The indicated amounts of cDNA were applied to real-time PCR. PCR performed without cDNA was used as a negative control (×) of the reaction. Data are presented as the number of PCR cycles to cross the threshold. Messenger RNA levels in these tissues were extrapolated from the PCR cycle of the liver for PPAR*α* or white adipose tissue for PPAR*γ* and then corrected by the chloramphenicol acetyltransferase cDNA content in each sample and presented in the manuscripts [13, 14].

Subsequently, the mRNA levels of PPARs *α* and *γ* were examined in mast cells. There were no significant PPAR*α* [13] and PPAR*γ* (our unpublished data) bands on Northern blot analysis of the RBL-2H3 mast cell line or of mouse bone marrow-derived mast cells (BMMCs). Then, PPAR*α* [13] and *γ* [14] mRNA levels in RBL-2H3 mast cell line were measured with the real-time semiquantitative polymerase chain reaction (PCR) and compared with levels in other organs. The PPAR*α* mRNA level is less than the level in 1000-times diluted liver, and the PPAR*γ* mRNA level is almost the same as the level in 100-times diluted white adipose tissue (Figure 3).

These observations that mast cells have very low levels of PPAR*α*/*γ* mRNA lead to another question: are these PPARs in mast cells effective?

Studies have examined whether fenofibrate (100 *μ*M) raises acyl-CoA oxidase mRNA levels, which are known to be induced by PPAR*α* activation [20, 21], and have shown that fenofibrate does not increase acyl-CoA oxidase mRNA levels in the RBL-2H3 mast cell line [13]. The effects of these PPAR*α* ligands on BMMCs from PPAR*α*-null mice were thoroughly examined, and both fenofibrate (100 *μ*M) and Wy-14,643 (30 *μ*M) were found to inihbit cysLT production [13]. It has been concluded that these compounds inhibit cysLT production independently of PPAR*α*.

We have observed that the immunoreactivity of anti-PPAR*γ* IgG in the RBL-2H3 mast cell line though ciglitazone (30 *μ*M) does not induce the mRNA level of acyl-CoA binding protein [18], which is a target gene of PPAR*γ* [22]. Diaz et al. [23] have examined PPAR*γ* protein in mouse BMMCs by SDS-PAGE immunoblot analysis and reported that the amount of PPAR*γ* in BMMCs is equivalent to that in the Jurkat T-cell line, which is known to have effective PPAR*γ* [24]. Maeyama et al. [25] have demonstrated that rosiglitazone (1–30 *μ*M) increases the proliferation of BMMCs, but that the proliferation is not observed in BMMCs from PPAR*γ* heterozygous deficient mice. Ward and Tan [26] have reviewed the contents of PPARs in various types of cells and have concluded that the PPAR*γ* in mast cells might play a role, and Paruchuri et al. [27] have recently reported that LTE_4_-induced COX-2 induction, prostaglandin D_2_ production, and ERK phosphorylation are sensitive for the interference of PPAR*γ* in the human mast cell sarcoma line LAD2 and may indicate a role of PPAR*γ* in mast cells. Further studies of the role of PPAR*γ* in mast cells are necessary.

## 5. WHAT IS THE TARGET?

The experimental findings that PPARs *α* and *γ* in mast cells seem not to be effective at very low mRNA levels lead to another question: what is the target of these compounds?

Fenofibrate (25 mg/kg p.o. for 10 days) induces proliferation of peroxisomes even in PPAR*α*-null mice [28]. Wy-14,643 (75 *μ*M) induces plasminogen activator inhibitor I with the induction of p38 and p42 mitogen-activated protein kinase (MAPK) phosphorylation 5 minutes after treatment, which would be too early for the induction to occur via transcription [29]. The ligand Wy-14,643 (1 *μ*M) leads to the phosphorylation of extracellular signal-regulated kinase (ERK) after 5 minutes of treatment but does not increase acyl-CoA oxidase mRNA levels [30].

The PPAR*γ* ligands ciglitazone (20 *μ*M) and 15-deoxy-Δ^12‐14^ prostaglandin J_2_(15 *μ*M) induce ERK, c-Jun N-terminal kinase, and p38 MAPK after 15 minutes of treatment, which might be earlier than transcription occurs [31]. The inducible effects of PPAR*γ* ligands on MAPK have been reported elsewhere [32, 33], and most authors have concluded that these effects are independent of PPAR*γ*.

MAPK is reported to induce 5-LOX activity in human polymorphonuclear cells and the Mono Mac 6 human monocytic leukemia cell line [34], and these findings may support the presence of PPAR-independent effects of PPAR *α* and *γ* ligands. However, MAPK phosphorylation has not been observed in mast cells treated with these PPAR ligands. The stimulating effect of these compounds on MAPK seems not to be the main mechanism of the PPAR-independent inhibition of cysLT production because it might increase the production of cysLTs.

The cysLT concentration is determined by subtracting degradation from production, and the PPAR-independent activation of MAPK increases cysLT production in mast cells. The degradation of cysLTs could be another mechanism of these drugs. The responsible enzymes of cysLT metabolism remain unclear. Recent findings that LTC_4_ is metabolized into LTD_4_ by *γ*-glutamyltransferase and *γ*-glutamylleukotrienase and that of double knockout mice of these enzymes do not metabolize LTC_4_ into LTD_4_ may indicate that these enzymes are the enzymes responsible for LTC_4_ degradation [35]. The degradation of LTD_4_ into LTE_4_ is reported to occur partly because of dipeptidase [36], but the responsible enzyme is still unclear. Induction of cytochrome P450 (CYP) 2B1/2 by phenobarbital in rats and the decrease in LTC_4_ concentrations in liver extract suggest the involvement of CYP2B1/2 in LTC_4_ degradation [37]. The CYP family comprises a large number of enzymes, and we do not yet have sufficient information on the contribution of CYP to cysLT metabolism.

Fujimura et al. [38] have reported that incubation with prostaglandin A_1_ (as PPAR*β*/*δ* ligand) and 15-deoxy-Δ^12‐14^ prostaglandin J_2_(as PPAR*γ* ligand) for more than 6 hours decreases the surface IgE receptor Fc *ε* RI in the KU812 human basophilic cell line, whereas LTB_4_ (as PPAR*α* ligand) does not. The PPAR*α* and *γ* ligands were preincubated for 2 hours before antigen treatment in mast cells [13, 17, 18], and the decrease of Fc *ε* RI on the surface of mast cells is not the main mechanism of the PPAR-independent inhibition of cysLT production. Regulation of the sensitivity to antigens is of pathological interest in allergic diseases, including asthma, and the interaction of mast cells with other inflammatory cells in pathological conditions should be examined.

## 6. CONCLUSION

These findings show that some effects of ligands of PPARs *α* and *γ* occur through a mechanism independent of PPARs *α* and *γ*. The involvement of PPARs *α* and *γ* should be examined in pharmacological experiments of PPAR ligands and of ligands of other nuclear receptors.

The involvement of PPAR*α* in the effects of PPAR ligands can be investigated in PPAR*α*-null mice [39] and at lower cost in mast cells, as described above.

PPAR*γ*-null mice die at 10.5 to 11.5 days post coitum because of placental dysfunction [40], and the contribution of PPAR*γ* cannot be examined in PPAR*γ*-homozygous knockout mice. One of the mutants of the PPAR*γ*2 subtype, ^Pro^12^Ala^, reduces transcription of wildtype tk-Luc-linked PPAR*γ*-related acyl-CoA oxidase, the peroxisome proliferator-responsible element, and lipoprotein lipase promoter by 40%, and persons homogenous for Ala-mutated PPAR*γ* have lower body mass indexes and higher serum levels of high-density lipoprotein cholesterol [41]. A 50% reduction in PPAR*γ* activity seems to have some biological effects, and PPAR*γ* heterozygous knockout mice, which are expected to have 50% lower levels of PPAR*γ* activity, and conditional knockout mice could be useful experimental models. Some RNA interference probes are available to inhibit PPAR*γ* transcription and would be useful tools for investigating PPAR*γ* involvement in cells, although the nonspecific interference by off-target effects should be noted.

Further investigations of the involvement of PPARs and other nuclear receptors in arachidonic acid metabolism are necessary to develop more effective and specific compounds as anti-asthma drugs.
